# Nasal Epithelial Cells Can Act as a Physiological Surrogate for Paediatric Asthma Studies

**DOI:** 10.1371/journal.pone.0085802

**Published:** 2014-01-27

**Authors:** Surendran Thavagnanam, Jeremy C. Parker, Michael E. McBrien, Grzegorz Skibinski, Michael D Shields, Liam G. Heaney

**Affiliations:** 1 Centre for Infection and Immunity, Queen's University Belfast, Belfast, Northern Ireland, United Kingdom; 2 Department of Anaesthetics, Royal Victoria Hospital, Belfast Health and Social Care Trust, Belfast, Northern Ireland, United Kingdom; 3 Department of Paediatrics, Royal Belfast Hospital for Sick Children, Belfast Health and Social Care Trust, Belfast, Northern Ireland, United Kingdom; 4 Paediatric Department, University Malaya Medical Centre, Kuala Lumpar, Malaysia; University of Houston, United States of America

## Abstract

**Introduction:**

Differentiated paediatric epithelial cells can be used to study the role of epithelial cells in asthma. Nasal epithelial cells are easier to obtain and may act as a surrogate for bronchial epithelium in asthma studies. We assessed the suitability of nasal epithelium from asthmatic children to be a surrogate for bronchial epithelium using air-liquid interface cultures.

**Methods:**

Paired nasal and bronchial epithelial cells from asthmatic children (n = 9) were differentiated for 28 days under unstimulated and IL-13-stimulated conditions. Morphological and physiological markers were analysed using immunocytochemistry, transepithelial-electrical-resistance, Quantitative Real-time-PCR, ELISA and multiplex cytokine/chemokine analysis.

**Results:**

Physiologically, nasal epithelial cells from asthmatic children exhibit similar cytokine responses to stimulation with IL-13 compared with paired bronchial epithelial cells. Morphologically however, nasal epithelial cells differed significantly from bronchial epithelial cells from asthmatic patients under unstimulated and IL-13-stimulated conditions. Nasal epithelial cells exhibited lower proliferation/differentiation rates and lower percentages of goblet and ciliated cells when unstimulated, while exhibiting a diminished and varied response to IL-13.

**Conclusions:**

We conclude that morphologically, nasal epithelial cells would not be a suitable surrogate due to a significantly lower rate of proliferation and differentiation of goblet and ciliated cells. Physiologically, nasal epithelial cells respond similarly to exogenous stimulation with IL-13 in cytokine production and could be used as a physiological surrogate in the event that bronchial epithelial cells are not available.

## Introduction

Airway epithelial cells not only provide a physical barrier to potentially harmful insults but they also play a significant role in the first line of immunological defence. There is increasing evidence that allergic diseases such as asthma are associated with epithelial disorders and, furthermore, that a primary abnormality of the airway epithelium may be central to disease causation [1], [2]. Chronic inflammation and airway remodelling are the main characteristics of asthma however they have been observed to occur in young children before asthma has become firmly established [3], [4]. In asthma, bronchial epithelial airway remodelling is characterised by goblet cell hyperplasia, reduced ciliated cell numbers and mucus hypersecretion [5], defective repair and proliferation [6], increased basal cell number [7] and impaired barrier function [8], [9].

Differentiated ALI cultures using the Transwell system have recently been shown to be an authentic model representing the airway epithelium *ex vivo* [10]. Differentiated ALI cultures from healthy subjects displayed a polarised pseudostratified multi-layered epithelium comprising basal, ciliated and goblet cells whereas cultures from asthmatic subjects displayed a dysfunctional epithelium consistent with the asthmatic airway *in vivo* [5]. The cultures closely mimic *in vivo* airway epithelial physiology in terms of cilia coverage and cilial beating, mucus production and formation of intact tight junctions [5], [11].

Samples from the lower airways in children can be obtained at the time of clinically indicated bronchoscopy but it is difficult to justify sampling the lower airways in otherwise healthy control children and in those with milder disease. The nasal epithelium therefore represents an attractive alternative although it is unclear how well the nasal epithelium represents the bronchial epithelium in paediatric asthma. According to the ‘united airway concept’ there is a close connection between the upper and lower airways [12]–[16]. This suggests that changes found in the nasal epithelium might mirror similar changes occurring in the lower airways. The link between allergic rhinitis and asthma has been underlined by epidemiological and clinical studies [12]–[16] which suggest that upper airway inflammation may reflect and provide an additional insight into lower airway involvement. Devalia *et al* have demonstrated that adult human nasal and bronchial epithelial cells cultured *in vitro* resemble the cells *in vivo* [17]. These studies involved using samples from adults and extrapolating data from adult studies cannot accurately represent the paediatric epithelium, especially since functional differences exist between adult and paediatric epithelium [18]. Several studies have shown similar morphology and response to cytokine stimulation between nasal and bronchial epithelium using submerged monolayer cultures, which only poorly represents the epithelium [19], [20]. Differentiated ALI cultures on the other hand are more authentic and can be analysed for markers of differentiation including MUC5AC (the major mucus-forming mucin) and SAM-pointed domain containing Ets-transcription factor (SPDEF) (a transcription factor in the goblet cell hyperplasia pathway) production as well as for goblet and ciliated cells and tight junction formation. As a result the differentiated ALI culture model is the most appropriate platform with which to conduct a comparison of paired nasal and bronchial differentiated ALI cultures.

In this study we aimed to compare the morphological and physiological profiles of nasal differentiated ALI cultures (PNECs) with bronchial differentiated ALI cultures (PBECs) under basal unstimulated and IL-13 stimulated conditions to determine the ability of PNECs to act as an *in vitro* surrogate for PBECs in asthma studies.

## Methods

### Subjects

Children less than 12 years of age (mean age 7.2 years [range: 1 to 12 years]) attending elective surgical procedures at the Royal Belfast Hospital for Sick Children were recruited. A doctor administered pro-forma was used to record the clinical history. Of the nine children with atopic asthma, defined as recurrent wheezing within the last year, 5 children had asthma plus allergic rhinitis and 4 children had asthma plus eczema but no allergic rhinitis (Table 1).

**Table 1:** Patient details including clinical status and clinical atopy.

Clinical Status	Clinical Atopy (assessed by questionnaire)	Serum IgE Concentration (kU/L)	Age (y)	Gender (M/F)	Treatment
Asthmatic	Allergic Rhinitis, Allergic Conjunctivitis	2258	6	M	ICS/LABA, LTA
Asthmatic	Eczema	154	7	M	ICS/LABA
Asthmatic	Eczema	446	4	F	SABA, when required
Asthmatic	Eczema, Allergic Rhinitis	5158	12	M	ICS/LABA
Asthmatic	Allergic Rhinitis	101	9	M	ICS/LABA
Asthmatic	Eczema	10	2	M	ICS, SABA
Asthmatic	Eczema	627	7	M	ICS/LABA
Asthmatic	Eczema, Allergic Rhinitis	100	11	M	ICS
Asthmatic	Allergic Rhinitis	40	1	M	ICS

Current treatment abbreviations are classed as follows: inhaled corticosteroid (ICS), long-acting beta agonist (LABA), short-acting beta agonist (SABA) and leukotriene antagonist (LTA).

### Ethics Statement

Written informed parental consent was obtained. This study was approved by the Office of the Research Ethics Committees of Northern Ireland (ORECNI).

### Isolation of PBECs and PNECs

Primary bronchial epithelial cells were obtained from asthmatic children as previously described [21]. Nasal brushings were performed by rotating an endocervical brush in each nostril. Asthmatic PBECs and asthmatic PNECs were cultured as previously described [5]. All brush washings were analysed for viruses using a multi-viral PCR analysis and only uncontaminated cultures were used [22].

### Differentiated ALI culture

ALI cultures (n = 9) were grown as previously described [5], [11]. Briefly, cells from subjects used in this study were grown at ALI at passage 2 for 28 days. At confluence, ALI was created by removing the apical medium and restricting the culture feeding to the basolateral compartment. The culture medium was changed on alternate days during which the cells differentiated over 28 days to ensure full differentiation as assessed by the presence of beating cilia and mucus on the apical surface of the cultures. We sampled 12 patients in total however 3 of the cultures either did not proliferate due to lack of cells from the initial sample or they did not form a 100% confluent monolayer prior to differentiation and so were rejected from the study.

### Stimulation of PBECs and PNECs with IL-13

Following the establishment of ALI, cells were fed basolaterally on alternate days with ALI medium [5] supplemented with recombinant human IL-13 (PeProTech EC Ltd, UK) at 20 ng/ml throughout the duration of the cultures (28 days) starting at day 0 ALI [11], [23]–[30].

### Transepithelial Electrical Resistance measurements (TEER)

We used TEER as a measure of ‘tight junction’ formation in epithelial cultures [31]. TEER was measured weekly using an EVOM meter (World Precision Instruments, FL, USA) [5], [11].

### Immunocytochemistry (ICC) for goblet and ciliated cell markers

Cytospin slides were made for the detection of goblet and ciliated cells from PBEC and PNEC cultures [5], [11]. Negative controls were subjected to routine conditions with the omission of the primary antibody. The results are expressed as the percentage differential goblet or ciliated cell count corrected for cell number from 3 slides per stain per insert per patient.

### RNA extraction and Quantitative Real-time PCR for MUC5AC and SPDEF mRNA

RNA extraction and cDNA synthesis were carried out using the RNeasy Mini kit (Qiagen, Crawley, UK) and First Strand cDNA Synthesis Kit (AMV) (Roche, UK) according to the manufacturer's protocol. Quantitative Real time PCR was carried out as previously described [11].

### Measurement of MUC5AC secreted apically using ELISA

Production of MUC5AC secreted mucin in the apical washes from PBECs and PNECs was measured as previously described [5], [11] using an adapted in-house MUC5AC ELISA [32]. We used mouse monoclonal antibody to MUC5AC [45M1] clone (Abcam, UK) as the primary antibody and Goat anti-mouse IgG HRP secondary antibody (Jackson Laboratories, USA) for detection of primary antibody binding. Results are expressed as the optical density corrected for MUC5AC positive control.

### Cytokine Analysis

Apical washings and basolateral supernatants from unstimulated and IL-13-stimulated PBECs and PNECs were analyzed using a 27-plex bead array assay (Bio-Plex Pro Human Cytokine 27-plex) (BioRad, UK) as per manufacturers' instructions. Results are only shown for analytes that were within the limits of detection (IL-1rα, IL-6, IL-7, IL-8, IL-12p70, granulocyte colony–stimulating factor (G-CSF), granulocyte macrophage colony-stimulating factor (GM-CSF), interferon gamma-induced protein 10 (IP-10), monocyte chemoattractant protein-1 (MCP-1), regulated upon activation, normal T cell expressed and secreted (RANTES) and vascular endothelial growth factor (VEGF).

### Statistical Analysis

Comparisons between PBECs and PNECs were made using paired t-tests and repeated measures ANOVA, with logarithmic transformation where appropriate. Cytokine concentrations were corrected for total cell number (pg/cell) and then the fold change in cytokine secretion following IL-13 stimulation was compared with unstimulated cytokine secretion (IL-13-stimulated/unstimulated). These results were then plotted on a log axis due to the varying ranges of all analytes involved. Individual cytokine profiles are included as supplemental data (Figures S1, S2, S3, S40, S5, S6) along with additional detail on all of the mentioned methods in the online data supplement (Methods S1).

## Results

It is unlikely given the number of passages the cells go through along with the length of time from sampling to completion of culture (7–10 weeks) that there would be any carry-over effect of inhaled corticosteriods in the following results.

### TEER

**Figure 1:**
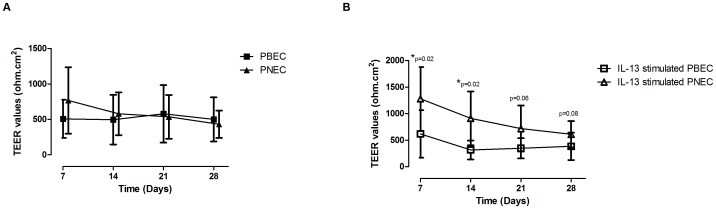
Measurement of Transepithelial Electrical Resistance (TEER). A - TEER of unstimulated PNECs and PBECs cultured for 28 d. Values are expressed as mean ± SD. No significant difference was observed in the TEER values between the groups. B - TEER of PNECs and PBECs cultured with 20 ng/ml IL-13 for 28 d. Values are expressed as mean ± SD. On days 7 and 14 there was a significant difference in TEER values between PNECs and PBECs (p<0.02 for each time point) however by the end of the culture period the TEER values were similar between groups.

We detected no overall differences in TEER values over time under unstimulated conditions between PBECs and PNECs [Figure 1A]. In contrast, following IL-13 stimulation, there was a significant difference in TEER values on days 7 (p = 0.02) & 14 (p = 0.02) between PBECs and PNECs with the difference in resistance becoming similar by days 21 & 28 [Figure 1B].

### Total Cell Count

On day 28 we found a significantly higher total cell number between PBECs [mean 5.7×10^5^ cells/ml (SD 1.2)] and PNECs [mean 3.4×10^5^ cells/ml (SD 0.8), (p = 0.002)] under unstimulated conditions [Table 2]. However there was no significant difference in the total cell number under IL-13 stimulated conditions [PBECs [mean 5.3×10^5^ cells/ml (SD 0.9); PNECs: mean 4.2×10^5^ cells/ml (SD 1.1)] [Table 2]. All subsequent results have been adjusted for total cell number.

**Table 2:** Total cell count on day 28 of ALI culture.

Sample	Mean number of cells (×10^5^ cells/ml)	Standard Deviation	P value	Treatment
PBEC(A)	5.7	1.2	0.002	Unstimulated
PNEC(A)	3.4	0.8		Unstimulated
PBEC(A)	5.3	0.9	>0.05	20 ng/ml IL-13
PNEC(A)	4.2	1.1		20 ng/ml IL-13

Under unstimulated conditions there were significant differences between PBECs and PNECs (p = 0.002). However under IL-13 stimulated conditions there were no significant differences between the same groups.

### Goblet Cell Quantification

**Figure 2:**
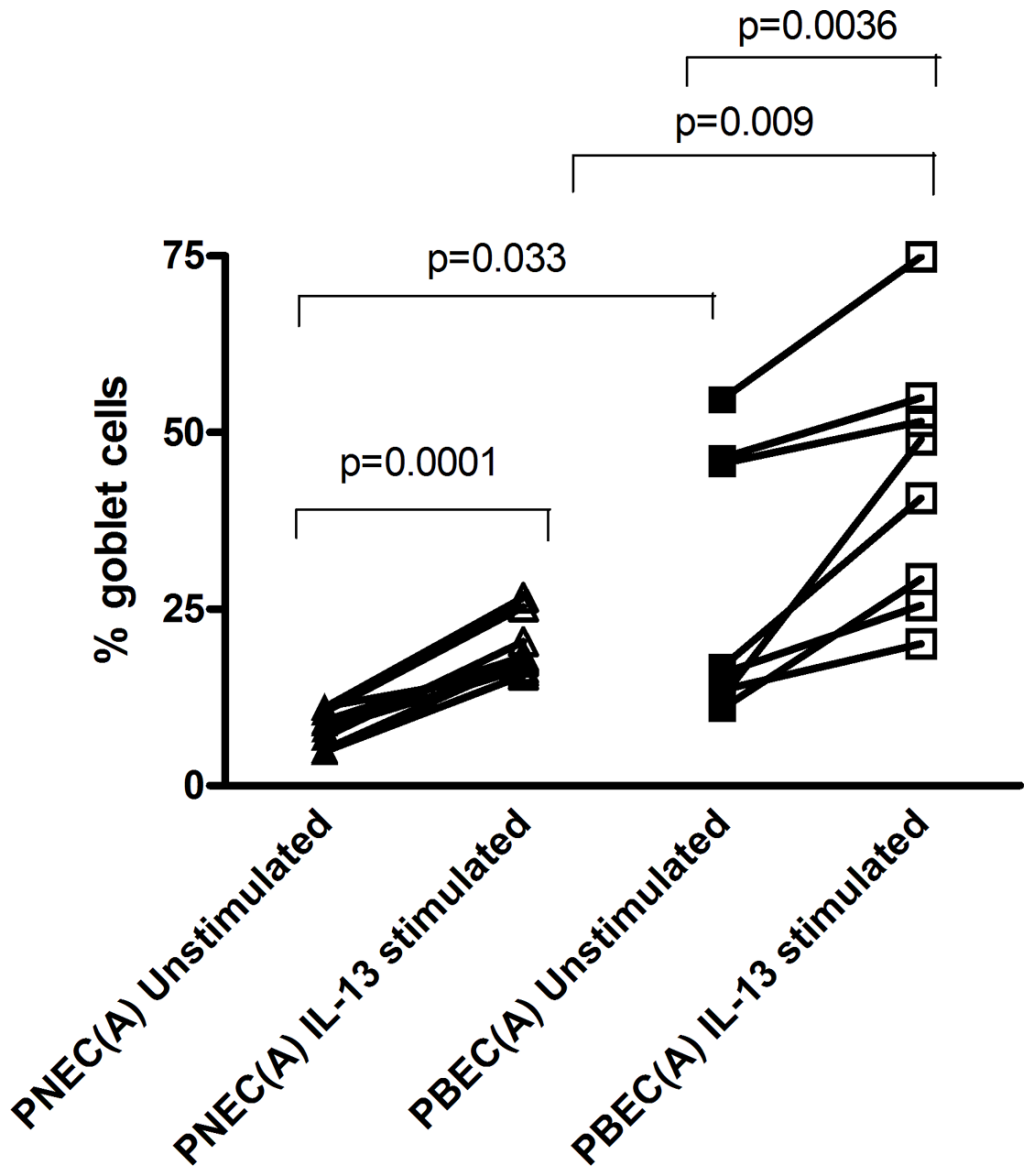
Quantification of Goblet Cell Number. Number of goblet cells from PNECs and PBECs treated with 20/ml IL-13 expressed as the percentage differential goblet cell count corrected for cell number on d 28 of ALI culture. Comparisons of average values between groups were performed using paired t-tests. There was a significant difference seen between unstimulated and IL-13 stimulated PNECs (p = 0.0001) and in IL-13 stimulated PBECs when compared to unstimulated PBECs (p = 0.0036). Additionally, there was a significant difference between PNEC unstimulated and PBEC unstimulated (p = 0.033) and when cells where stimulated with IL-13, the percentage of goblet cells was significantly higher in stimulated PBECs compared to stimulated PNECs (p = 0.009).

IL-13 stimulation resulted in a similar increase in the percentage of goblet cells in both PBECs [43.3% (SD 18.1), p = 0.0036] and PNECs [19.4% (SD 4.0), p = 0.0001] compared with unstimulated controls [PBEC: 27.1% (SD 18.4); PNEC: 8.4% (SD 2.4)] [Figure 2]. In addition there was a significant difference in goblet cell percentage between PNEC unstimulated and PBEC unstimulated [8.4% (SD 2.4) versus 27.1% (SD 18.4) respectively, p = 0.033] and PNEC IL-13 stimulated and PBEC IL-13 stimulated [19.4% (SD 4.0) versus 43.3% SD (18.1) respectively, p = 0.009].

### Real Time PCR

**Figure 3:**
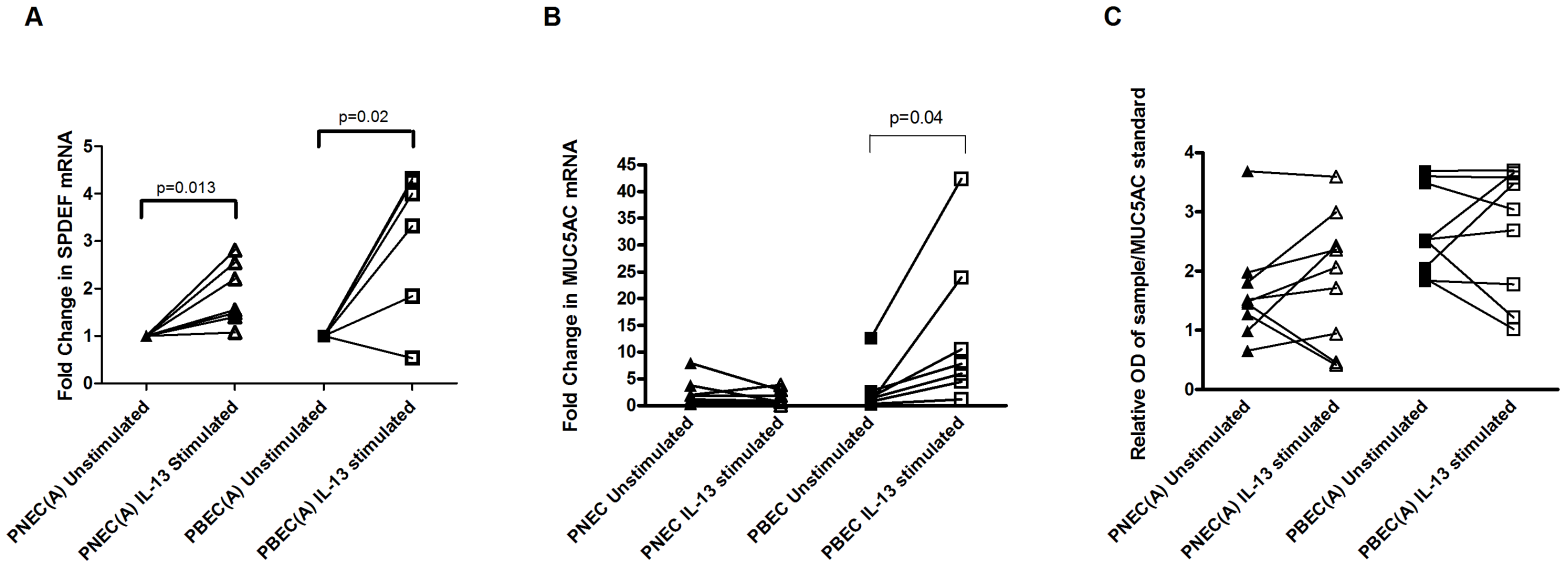
Gene expression of SPDEF & MUC5AC mRNA and ELISA for MUC5AC mucin secretion. A - Gene expression of SPDEF mRNA using comparative quantitation real time PCR in IL-13 stimulated PNECs and PBECs expressed as fold change compared to unstimulated cells. In both cell types, stimulation with IL-13 caused a significant increase in SPDEF mRNA levels (PNECs: p = 0.013; PBECs: p = 0.02). B - Gene expression of MUC5AC mRNA using comparative quantitation real time PCR in IL-13 stimulated PNECs and PBECs expressed as fold change compared to unstimulated cells. There was no significant increase in MUC5AC mRNA levels in PNECs, however, stimulation with IL-13 caused a significant increase in MUC5AC mRNA levels in PBECs (p = 0.04). C - Relative optical density (OD λ = 450 nm) of MUC5AC secreted apically using ELISA corrected for MUC5AC positive control. There was no significant difference between unstimulated and IL-13 stimulated PNECs or PBECs.

SPDEF mRNA was higher in IL-13 stimulated PNECs (p = 0.013) and IL-13 stimulated PBECs (p = 0.02) compared with unstimulated PNECs and PBECs demonstrating a similar response to exogenous stimulation between PBECs and PNECs [Figure 3A]. However under IL-13 stimulation there was a similar level of MUC5AC mRNA between stimulated and unstimulated PNECs whereas IL-13 stimulated PBECs had higher levels of MUC5AC mRNA than unstimulated PBECs (p = 0.04) [Figure 3B].

### Measurement of MUC5AC secreted apically using ELISA

Under IL-13 stimulation similar quantities of apically secreted mucin were measured between PNECs and PBECs [Figure 3C].

### Ciliated Cell Quantification

**Figure 4:**
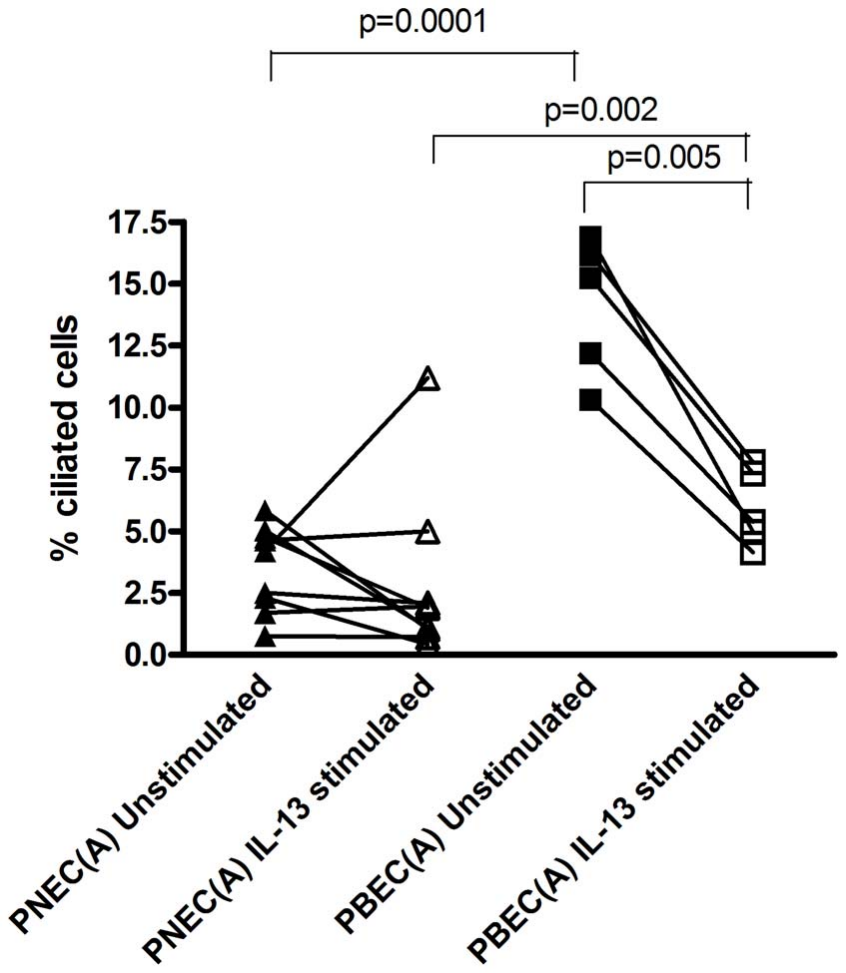
Quantification of Ciliated Cells. Number of ciliated cells from PNECs and PBECs treated with 20/ml IL-13 expressed as the percentage differential ciliated cell count corrected for cell number on d 28 of ALI culture. Comparisons of average values between groups were performed using paired t-tests. In unstimulated conditions, PBEC cultures contained a significantly higher percentage of ciliated cells than PNECs (p = 0.0001). However, when stimulated with IL-13, the percentage of ciliated cells significantly decreased in PBECs (p = 0.005), but IL-13 had no effect on ciliated cell numbers in PNECs. Additionally, IL-13 stimulated PBECs had significantly higher numbers of ciliated cells compared with IL-13 stimulated PNECs (p = 0.002).

IL-13 stimulated PBECs had lower ciliated cell numbers than unstimulated PBECs [5.9% (SD 1.6) versus 14.8% (SD 2.5) respectively, p = 0.005] which was not observed between PNEC IL-13 stimulated and PNEC unstimulated [2.8% (SD 3.4) versus 3.5% (SD 1.7) respectively] [Figure 4]. There was a significant difference between PBEC unstimulated and PNEC unstimulated [14.8% (SD 2.5) versus 3.5% (SD 1.7) respectively, p = 0.0001] [Figure 4]. In addition there was a significant difference between PBEC IL-13 stimulated and PNEC IL-13 stimulated [5.9% (SD 1.6) versus 2.8% (SD 3.4) respectively, p = 0.002] [Figure 4].

### Cytokine production from PBECs and PNECs

**Figure 5:**
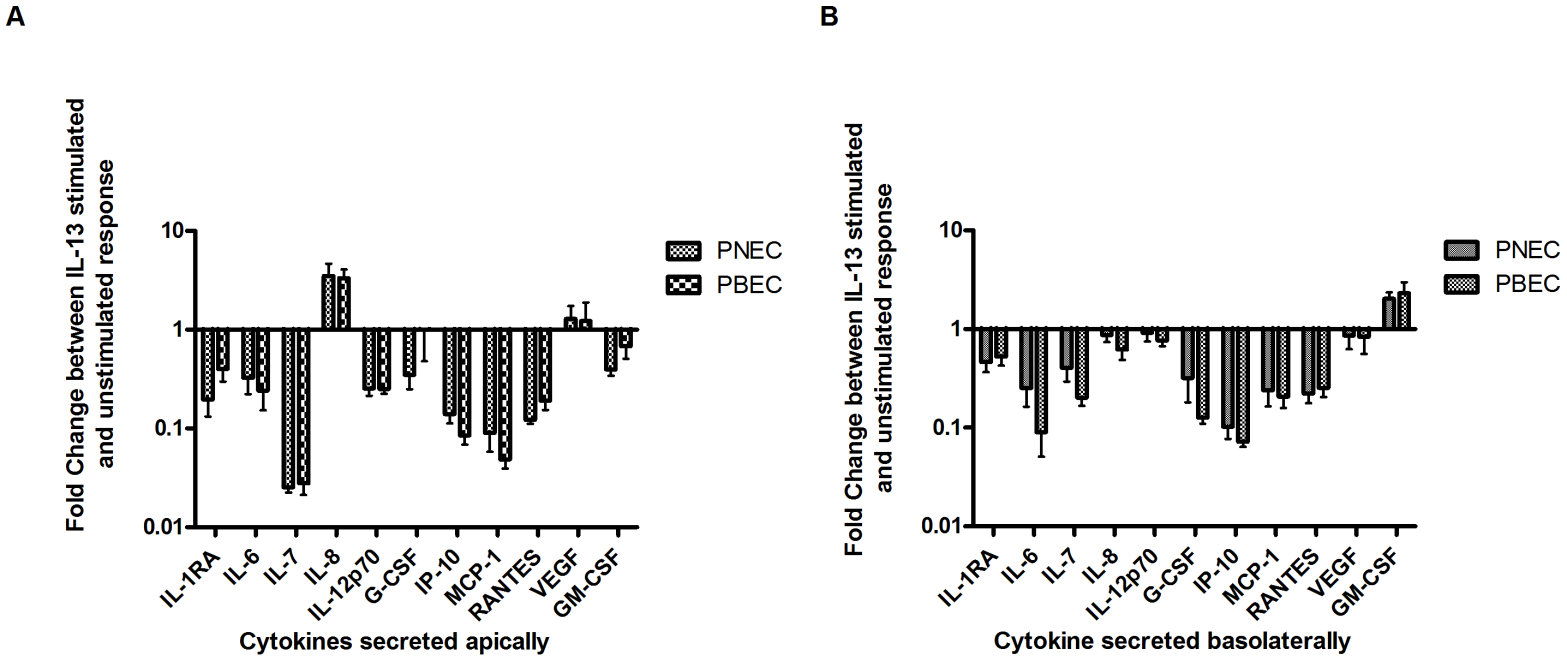
Cytokine Analysis of Apical Washings and Basolateral Supernatants using Bioplex. A – Apical secretion of cytokines in PBEC and PNEC cultures. Results were corrected for cell numbers and a fold change ratio was calculated (IL-13 stimulated/unstimulated). In order to graphically represent all detectable cytokines the results were plotted on a logarithmic axis. There were no significant differences in any of the detected cytokines secreted between PNECs and PBECs. B - Basolateral secretion of cytokines in PBEC and PNEC cultures. Results were corrected for cell number and a fold change ratio was then calculated (IL-13 stimulated/unstimulated). In order to graphically represent all detectable cytokines the results were plotted on a logarithmic axis. There were no significant differences in any of the cytokines secreted between PNECs and PBECs.

Analytes that were within the limits of detection were IL-1rα, IL-6, IL-7, IL-8, IL-12p70, granulocyte colony–stimulating factor (G-CSF), granulocyte macrophage colony-stimulating factor (GM-CSF), interferon gamma-induced protein 10 (IP-10), monocyte chemoattractant protein-1 (MCP-1), regulated upon activation, normal T cell expressed and secreted (RANTES) and vascular endothelial growth factor (VEGF). The fold change of apically secreted cytokines after IL-13 stimulation [Figure 5A] showed there to be no significant difference between PBECs and PNECs. Similarly, there was no significant difference in basolaterally secreted cytokines between PBECs and PNECs stimulated with IL-13 [Figure 5B] demonstrating an overall similar response to IL-13 stimulation between PNECs and PBECs. Individual cytokine secretion graphs are included in the supplementary data section of this paper.

## Discussion

Nasal epithelial cells provide an attractive alternative to bronchial epithelial cells for use in asthma research due to their accessibility without the need for anaesthesia and intubation however it is unclear whether differentiated nasal ALI cultures represent the differentiated bronchial ALI cultures morphologically and physiologically. We have compared paired nasal and bronchial epithelial cells differentiated at ALI from asthmatic children in order to determine whether nasal epithelial cells can act as a surrogate for bronchial epithelial cells. We have found that PNECs exhibit different morphological features such as lower proliferation rates and differentiation of goblet and ciliated cells compared with PBECs however their physiological response of secreted cytokines to exogenous stimulation with IL-13, a key cytokine in the pathogenesis of asthma is strikingly similar when corrected for cell number.

In considering morphology, unstimulated PNECs do not exhibit the constitutive goblet cell hyperplasia seen in unstimulated PBECs [5]. This is to be expected as asthma is a lower airways disease. Mouse models of allergic rhinitis have reported that a goblet cell hyperplasia may only exist following stimulation with ovalbumin [33], [34] or TGF-β [35]. IL-13 has been shown to increase goblet cell hyperplasia in non-asthmatic and asthmatic paediatric bronchial epithelium [11] and in this study we have shown that PNECs also respond with a goblet cell increase to IL-13 stimulation along with a significant increase in SAM-pointed domain-containing Ets-like factor (SPDEF) mRNA which is implicated in the goblet cell hyperplasia pathway [36]. Miyahara and colleagues found that although IL-13 is a major contributor to the late nasal response it did not induce a goblet cell hyperplasia [37] however our data is at odds with this finding. The IL-13 mediated reduction of ciliated cells in PBECs is not observed with IL-13 stimulation in PNECs however.

In a previous study by McDougall *et al.* which compared bronchial and nasal epithelial monolayers from adults and children they found the cells to look morphologically similar [19]. However, differentiated nasal ALI cultures display notable morphological differences when compared with the differentiated bronchial ALI cultures which could not have been detected in submerged monolayer cultures. We found that PNECs did not proliferate or differentiate at the same rate as PBECs with a significant lower total cell number at the end of the culture period in addition to the differences seen in goblet and ciliated cells under basal and stimulated conditions. PBECs appeared to exhibit consistent distinct morphological changes in response to IL-13 stimulation whereas PNECs responded in a variable manner. We did however observe some similarities between PBECs and PNECs: TEER measurements, which is at odds with the TEER data generated by Lopez-Souza and colleagues [38] and MUC5AC secretion under unstimulated conditions. Taken together, morphologically there does not appear to be enough consistency between PBECs and PNECs which would suggest that for studies focussing on the morphology of the asthmatic bronchial epithelium, PNECs would not serve as an appropriate surrogate.

In considering physiological response we examined the effects of an exogenous cytokine, namely IL-13, on cytokine/mediator release from PBECs and PNECs and found a similar response profile when corrected for cell number. McDougall *et al.* found that constitutive and cytokine-stimulated release of particular cytokines (IL-6, IL-8, MMP-9 and RANTES) was significantly higher in nasal monolayer than bronchial monolayer cultures but that on further analysis nasal and bronchial epithelial cells responded in the same way showing significant correlation [19]. In a follow-up study from the same group, looking specifically at mediator release between nasal and bronchial epithelial monolayer cultures from children, Pringle and colleagues found that RANTES, MMP-9, TIMP-1, MCP-1 and VEGF were similar between nasal and bronchial monolayer cultures whereas IL-6, IL-8 and G-CSF were higher [20]. The difference in observations of MMP-9 and RANTES between the studies is proposed to be due to the first study having a mixed population of adult and paediatric epithelial cells. We demonstrated a similar finding to McDougall *et al.* and Pringle *et al.* in those cytokines from our panel that were detectable. Both PNECs and PBECs responded to IL-13 stimulation (corrected for cell numbers) in a similar manner, albeit with PNECs generally expressing higher levels of cytokine. This would suggest that physiologically for the cytokines measured, PNECs could be used as a surrogate for PBECs. Further studies would hope to confirm this observation however it would suggest that the similarities seen in monolayer mediator release are maintained throughout the differentiation process.

In a more recent study using well-differentiated paediatric asthmatic and non-asthmatic paired bronchial and nasal epithelial cells, Lopez-Guisa and colleagues also suggested that there was correlation in mediator release between PBEC and PNEC cultures [39]. This group showed TGF-β2 and VEGF to be significantly increased in asthmatic bronchial and nasal epithelium compared to non-asthmatic under unstimulated conditions. TGF-β2 and periostin were significantly up-regulated in nasal and bronchial asthmatic epithelium under unstimulated and following IL-4/IL-13 stimulation [39]. Unfortunately our study did not have a sample of non-asthmatic children to perform a similar comparison. However, our study broadly agrees with Lopez-Guisa *et al.* in terms of the similarities observed in mediator release between nasal and bronchial asthmatic epithelium.

IL-13 resulted in the reduction of the majority of cytokines, with the exceptions of IL-8 and VEGF, when secreted apically and basolaterally and GM-CSF when secreted basolaterally. Surprisingly, a number of cytokines that we expected to increase actually decreased including IL-1ra, GM-CSF and MCP-1 [40]–[43]. While there are various studies that have noted increases in these cytokines under stimulation with IL-13 we would add that many used different culture systems, stimulation concentrations and treatment regimens compared to our study, which may go some way to explaining the unexpected decrease in these mediators. We believe this is a true reflection of the epithelium as in both PBECs and PNECs we saw strikingly similar responses to our chronic stimulation.

Our data confirm that characteristics and responses in submerged monolayer cultures differ from differentiated ALI cultures. We believe that our differentiated ALI model authentically represents the true state of the epithelium *in vivo* [10]. A recent study by Ogilvie and colleagues, who compared paired CF nasal and bronchial epithelial brushings using microarray, found that differences in the global gene expression profiles of CF and non-CF nasal and bronchial epithelial cells existed. They recommended not using nasal epithelial cells as a surrogate for pre-screening prior to lung-directed therapies [44]. Whether nasal epithelium should be used in place of bronchial epithelium will inevitably be determined by the focus and design of future studies and with a growing literature on the many comparisons now taking place, we will be much better informed of the benefits or drawbacks of using nasal as a surrogate for bronchial epithelial cells.

Our study was limited in a number of ways. Firstly, we were unable to sample a large enough number of non-asthmatic controls to perform a comparison between health and disease. Secondly, in terms of stimulation only one cytokine (IL-13) was used due to the quantity of cells available. It would be interesting to know if other stimuli in PNECs reflect the response in PBECs. Additional studies are required to further explore the use of nasal epithelial cells as a surrogate.

In conclusion, we have demonstrated that our models of differentiated PNECs and PBECs display notable morphological differences which would question the use of PNECs as a reliable and reproducible morphological surrogate, especially in asthma where characteristic traits such as constitutive goblet cell hyperplasia are absent in PNECs from asthmatics. However, we have also demonstrated that physiologically, both PNECs and PBECs respond in the same way to stimulation with IL-13 suggesting that PNECs could be used as a physiological surrogate for PBECs in asthmatic studies in the event that bronchial epithelial cells are not available.

## Supporting Information

Figure S1 **Cytokine analysis of IL-1ra, IL-6, IL-7 & IL-8 secreted apically.** A - Analysis of IL-1ra secreted apically using the Bioplex system. Results were corrected for cell number and absolute concentrations plotted in pg/cell. IL-13 stimulated PNECs and PBECs secreted significantly less IL-1ra compared with unstimulated PNECs (p = 0.02) and PBECs (p = 0.001). Additionally unstimulated PNECs secreted higher levels of IL-1ra compared with unstimulated PBECs (p = 0.04). B - Analysis of IL-6 secreted apically using the Bioplex system. Results were corrected for cell number and absolute concentrations plotted in pg/cell. IL-13 stimulated PNECs and PBECs secreted significantly less IL-6 compared with unstimulated PNECs (p = 0.02) and PBECs (p = 0.01). Additionally unstimulated PNECs secreted higher levels of IL-6 compared with unstimulated PBECs (p = 0.04). C - Analysis of IL-7 secreted apically using the Bioplex system. Results were corrected for cell number and absolute concentrations plotted in pg/cell. IL-13 stimulated PNECs and PBECs secreted significantly less IL-7 compared with unstimulated PNECs (p = 0.0003) and PBECs (p = 0.0004). Additionally unstimulated PNECs secreted higher levels of IL-7 compared with unstimulated PBECs (p = 0.04). D - Analysis of IL-8 secreted apically using the Bioplex system. Results were corrected for cell number and absolute concentrations plotted in pg/cell. IL-13 stimulated PNECs and PBECs secreted higher levels of IL-8 compared with unstimulated PNECs and PBECs however they did not reach statistical significance. Additionally IL-13 stimulated PNECs secreted higher levels of IL-8 compared with IL-13 stimulated PBECs (p = 0.03).
(TIF)

Figure S2 **Cytokine analysis of IL-12p70, G-CSF, IP-10 & MCP-1 secreted apically.** A - Analysis of IL-12p70 secreted apically using the Bioplex system. Results were corrected for cell number and absolute concentrations plotted in pg/cell. IL-13 stimulated PNECs and PBECs secreted significantly less IL-12p70 compared with unstimulated PNECs (p = 0.007) and PBECs (p = 0.0009). Additionally unstimulated PNECs secreted higher levels of IL-12p70 compared with unstimulated PBECs although this was not statistically significant (p = 0.08). B - Analysis of G-CSF secreted apically using the Bioplex system. Results were corrected for cell number and absolute concentrations plotted in pg/cell. IL-13 stimulated PNECs secreted significantly less G-CSF compared with unstimulated PNECs (p = 0.03). There was no significant difference between IL-13 stimulated and unstimulated PBECs. Additionally unstimulated PNECs secreted higher levels of G-CSF compared with unstimulated PBECs although this was not statistically significant. C - Analysis of IP-10 secreted apically using the Bioplex system. Results were corrected for cell number and absolute concentrations plotted in pg/cell. IL-13 stimulated PNECs and PBECs secreted lower levels of IP-10 compared with unstimulated PNECs (p = 0.006) and PBECs (p = 0.01). Additionally unstimulated PNECs secreted significantly higher levels of IP-10 compared with unstimulated PBECs (p = 0.047) and IL-13 stimulated PNECs secreted higher levels of IP-10 compared with IL-13 stimulated PBECs (p = 0.04). D - Analysis of MCP-1 secreted apically using the Bioplex system. Results were corrected for cell number and absolute concentrations plotted in pg/cell. IL-13 stimulated PNECs and PBECs secreted lower levels of MCP-1 compared with unstimulated PNECs (p = 0.005) and PBECs (p = 0.01). Additionally unstimulated PNECs secreted significantly higher levels of IP-10 compared with unstimulated PBECs (p = 0.001).
(TIF)

Figure S3 **Cytokine analysis of RANTES, VEGF & GM-CSF secreted apically.** A - Analysis of RANTES secreted apically using the Bioplex system. Results were corrected for cell number and absolute concentrations plotted in pg/cell. IL-13 stimulated PNECs and PBECs secreted lower levels of RANTES compared with unstimulated PNECs (p = 0.003) and PBECs (p = 0.049). Additionally unstimulated PNECs secreted significantly higher levels of RANTES compared with unstimulated PBECs (p = 0.047). B - Analysis of VEGF secreted apically using the Bioplex system. Results were corrected for cell number and absolute concentrations plotted in pg/cell. There was no significant difference between IL-13 stimulated PNECs and PBECs compared with unstimulated PNECs and PBECs or between PNECs and PBECs. C - Analysis of GM-CSF secreted apically using the Bioplex system. Results were corrected for cell number and absolute concentrations plotted in pg/cell. IL-13 stimulated PNECs secreted lower levels of GM-CSF compared with unstimulated PNECs (p = 0.009). There was no significant change in GM-CSF between IL-13 stimulated and unstimulated PBECs. Additionally unstimulated PNECs secreted significantly higher levels of GM-CSF compared with unstimulated PBECs (p = 0.01).
(TIF)

Figure S4 **Cytokine analysis of IL-1ra, IL-6, IL-7 & IL-8 secreted basolaterally.** A - Analysis of IL-1ra secreted basolaterally using the Bioplex system. Results were corrected for cell number and absolute concentrations plotted in pg/cell. IL-13 stimulated PNECs secreted significantly less IL-1ra compared with unstimulated PNECs (p = 0.04). There was no significant difference between IL-13 stimulated and unstimulated PBECs. Additionally there was no difference in IL-1ra secretion between PNECs and PBECs. B - Analysis of IL-6 secreted basolaterally using the Bioplex system. Results were corrected for cell number and absolute concentrations plotted in pg/cell. IL-13 stimulated PNECs secreted less IL-6 compared with unstimulated PNECs however, this did not reach statistical significance. There was a significant difference in IL-6 secretion between IL-13 stimulated and unstimulated PBECs (p = 0.04). Additionally the IL-13 stimulated PNECs secreted higher levels of IL-1ra compared with IL-13 stimulated PBECs (p = 0.04). C - Analysis of IL-7 secreted basolaterally using the Bioplex system. Results were corrected for cell number and absolute concentrations plotted in pg/cell. IL-13 stimulated PNECs and PBECs secreted less IL-7 compared with unstimulated PNECs (p = 0.06) and PBECs (p = 0.05) but these did not reach statistical significance. Additionally there was no significant difference in IL-7 secretion between PBECs and PNECs. D - Analysis of IL-8 secreted basolaterally using the Bioplex system. Results were corrected for cell number and absolute concentrations plotted in pg/cell. There was no significant difference in IL-8 secretion between IL-13 stimulated PNECs and PBECs compared with unstimulated PNECs and PBECs or between PNECs and PBECs.
(TIF)

Figure S5 **Cytokine analysis of IL-12p70, G-CSF, IP-10 & MCP-1 secreted basolaterally.** A - Analysis of IL-12p70 secreted basolaterally using the Bioplex system. Results were corrected for cell number and absolute concentrations plotted in pg/cell. There was no significant difference between IL-13 stimulated PNECs and PBECs compared with unstimulated PNECs and PBECs. However unstimulated PNECs secreted higher levels of IL-12p70 compared with unstimulated PBECs (p = 0.009). B - Analysis of G-CSF secreted basolaterally using the Bioplex system. Results were corrected for cell number and absolute concentrations plotted in pg/cell. There was no significant difference between IL-13 stimulated PNECs and PBECs compared with unstimulated PNECs and PBECs although there was a large decrease in concentration. However IL-13 stimulated PNECs secreted higher levels of IL-12p70 compared with IL-13 stimulated PBECs (p = 0.048). C - Analysis of IP-10 secreted basolaterally using the Bioplex system. Results were corrected for cell number and absolute concentrations plotted in pg/cell. There was no significant difference between IL-13 stimulated PNECs and PBECs compared with unstimulated PNECs and PBECs. However unstimulated PNECs secreted higher levels of IP-10 compared with unstimulated PBECs (p = 0.009). D - Analysis of MCP-1 secreted basolaterally using the Bioplex system. Results were corrected for cell number and absolute concentrations plotted in pg/cell. There was lower MCP-1 secretion between IL-13 stimulated PNECs and PBECs compared with unstimulated PNECs and PBECs however this did not reach statistical significance. Unstimulated PNECs secreted significantly higher levels of MCP-1 compared with unstimulated PBECs (p = 0.003) as did IL-13 stimulated PNECs compared with IL-13 stimulated PBECs which was close to reaching significance (p = 0.05).
(TIF)

Figure S6 **Cytokine analysis of RANTES, VEGF & GM-CSF secreted basolaterally.** A - Analysis of RANTES secreted basolaterally using the Bioplex system. Results were corrected for cell number and absolute concentrations plotted in pg/cell. There was no significant difference between IL-13 stimulated PNECs and PBECs compared with unstimulated PNECs and PBECs or between PNECs and PBECs. B - Analysis of VEGF secreted basolaterally using the Bioplex system. Results were corrected for cell number and absolute concentrations plotted in pg/cell. There was no significant difference between IL-13 stimulated PNECs and PBECs compared with unstimulated PNECs and PBECs or between PNECs and PBECs. C - Analysis of GM-CSF secreted basolaterally using the Bioplex system. Results were corrected for cell number and absolute concentrations plotted in pg/cell. IL-13 stimulated PNECs secreted significantly more GM-CSF compared with unstimulated PNECs (p = 0.039). Additionally there was no significant difference in GM-CSF secretion between IL-13 stimulated and unstimulated PBECs or between PBECs and PNECs.
(TIF)

Methods S1 **Full descriptions of each method used in the study are provided in the supplementary methods section.**
(DOC)
