# Supplementary material for: Nasal Epithelial Cells Can Act as a Physiological Surrogate for Paediatric Asthma Studies
Source: PLoS One. 2014 Jan 27;9(1):e85802. doi: 10.1371/journal.pone.0085802 (PMC3903489; doi:10.1371/journal.pone.0085802)
Supplement: Methods S1 — Full descriptions of each method used in the study are provided in the supplementary methods section. (DOC) [file pone.0085802.s007.doc]

**Nasal epithelial cells can act as a physiological surrogate for paediatric asthma studies**

Surendran Thavagnanam^1^*, Jeremy C Parker^1^*, Michael E McBrien^2^, Grzegorz Skibinski^1^, Liam G Heaney^1^, Michael D Shields^1, 3^

**Online Data Supplement**

**Methods**

**Subjects**

Children less than 12 years of age (range: 1 to 12 years) attending elective surgical procedures at the Royal Belfast Hospital for Sick Children were recruited. A doctor administered semi-structured pro-forma was used to record the clinical history. All asthmatic children were treated with a regular inhaled corticosteroid using rescue short acting beta-agonist therapy as and when required for symptoms. Patient details are listed in Table 1. Written informed parental consent and where appropriate child assent was obtained. This study was approved by the Office of the Research Ethics Committees of Northern Ireland (ORECNI).

**Isolation of primary paediatric bronchial (PBECs) and nasal (PNECs) epithelial cells**

Non-bronchoscopic paired bronchial brushings and nasal brushings were obtained from asthmatic children (n=9) as previously described [21]. Nasal brushings were performed by rotating an endocervical brush in each nostril. Briefly, the cells obtained from the bronchial and nasal brushes were removed and centrifuged. The pellet was then seeded into a collagen coated T10 flask and allowed to grow until the cells were 80% confluent before transferring them to a collagen coated T75 flask, and finally onto collagen coated transwells at 0.8x10^5^ cells per well seeding density. PBECs and PNECs were cultured using methods developed in our laboratory which resulted in homogeneous cultures of basal epithelial cells [5]. Bronchial brush washings were analysed for viruses using a multi-viral PCR analysis [22] and only uncontaminated cultures were used.

**Differentiated ALI culture**

ALI cultures were grown as previously described [5,11]. All cells from subjects used in this study were grown at ALI at passage 2. The cells were grown submerged in transwells for the first 9 to 14 days, during which time the culture medium was changed on day 1 and every other day thereafter. Once the cells reached 100% confluence, an ALI was created by removing the apical medium and restricting the culture feeding to the basolateral compartment. Following ALI establishment, the culture medium was changed on alternate days and the cells were allowed to grow at ALI for 28 days to ensure full differentiation as assessed by the presence of beating cilia and mucus on the apical surface of the cultures.

**Stimulation of PBECs and PNECs with IL-13**

Following the establishment of ALI, cells were fed basolaterally every other day with ALI medium supplemented with recombinant human IL-13 (PeProTech EC Ltd, London, UK) at 20ng/ml in line with previous studies which have used concentrations ranging from 0.1ng/ml to 100ng/ml [11, 23-30]. The apical surface was washed weekly with phosphate buffered saline (PBS) and samples stored at -80°C for further analysis.

**Transepithelial Electrical Resistance measurements (TEER)**

We used TEER as a measure of ‘tight junction’ formation in epithelial cultures [31]. TEER was measured on days 7, 14, 21 and 28 of ALI culture using an EVOM meter (World Precision Instruments, FL, USA). The TEER of six inserts per treatment group was measured at each time point and the average was calculated. Briefly, the apical surface was washed prior to measurement with warm PBS (500 µl). Following aspiration, warm DMEM with no additives was applied apically followed by the TEER being measured. The DMEM was then removed, and the cultures were fed as per normal.

**Immunocytochemistry (ICC) for goblet and ciliated cell markers**

Cytospin slides were made to allow for the detection of MUC5AC-positive (goblet) and acetylated alpha-tubulin-positive (ciliated) cells from the PBEC and PNEC cultures as previously described [5, 11]. Immediately after trypsinisation, cytospin slides were prepared with a cell density of 5.0 x 10^4^ cells per slide. Immunostaining was performed using mouse monoclonal anti-MUC5AC antibody (1:100) [Abcam, UK] and mouse monoclonal acetylated alpha tubulin antibody (1:700) [Abcam, UK] for goblet and ciliated cells respectively. The reaction between the antigen and the antibody was detected using a perixodase-conjugated goat anti-mouse secondary antibody (Vector Laboratories, Peterborough, UK). The slides were then stained with DAB substrate kit (Vector Laboratories, Peterborough, UK) and counterstained with haematoxylin (Sigma-Aldrich, UK) for visualization. Negative controls were subjected to routine conditions with the omission of the primary antibody. MUC5AC and acetylated alpha tubulin positive cells were counted (500 cells/slide) at a magnification of x 40 blindly in 6 fields of vision and are expressed as the percentage differential goblet or ciliated cell count corrected for cell number from 3 slides per stain per insert.

**RNA extraction and Quantitative Real-time PCR for MUC5AC and SPDEF mRNA**

RNA extraction was carried out using RNeasy Mini kit (Qiagen, Crawley, UK) according to the manufacturer’s instructions and quantified on a spectrophotometer. First Strand cDNA Synthesis Kit for RT-PCR (AMV) (Roche, UK) was then used according to the manufacturer’s protocol. For the real time PCR reaction, the DNA amplification was carried out using the Fast Start Universal SYBR Green Master (Rox) (Roche, UK) according to the manufacturers’ protocol. For each PCR reaction separate PCR Master Mixes were prepared containing GAPDH (Tebu-bio, Peterborough, UK), MUC5AC or SPDEF primers. MUC5AC and SPDEF primers sequences are as follows (Invitrogen, Paisley, UK):

**MUC5AC**

Forward 5’ TCC TTT CGT GTT GTC ACC GA 3’

Reverse 5’ TCT TGA TGG CCT TGG AGC 3’

**SPDEF**

Forward 5’ AGC CTA CAG AAG GGC AGT GA 3’

Reverse 5’ AAC TCA GGG GTG CAG ATG TC 3’

A final total volume of 10μl was constituted with the addition of 1μl of cDNA added into each mastermix. Samples were placed in individual wells in triplicate on a Thermo-Fast 96-well detection plate and run on the spectrofluorometric thermal cycler AB 7000 (ABI Prism).

**Measurement of MUC5AC secreted apically using ELISA**

Production of MUC5AC secreted mucin in the apical washes from PBECs and PNECs was measured using an in-house MUC5AC ELISA adapted from Takeyama and colleagues [32]. As no MUC5AC standard was commercially available, this ELISA produced a semi-quantitative analysis of the production of MUC5AC. A 96-well high-binding ELISA plate (Fisher Scientific, UK) was coated with experimental samples at 37^o^C overnight. The plate was then washed three times with PBS. Wells were blocked with a solution of 2% BSA in PBS for 1 hour at room temperature. Following three washes with PBS, samples were exposed to primary antibody diluted 1:200 in PBS containing 0.05% Tween®20 (Sigma-Aldrich, Dorset, UK). After incubating for 1 hour, the plate was washed three times and labelled with an HRP-conjugated goat anti-mouse IgG (Jackson Laboratories, USA) diluted to 1:10,000 in PBS + 0.05% Tween®20 and incubated for 1 hour. After the secondary antibody incubation, the plate was washed three times with PBS, and the colour reaction was developed with 100μl of TMB (Millipore, UK) per well incubated for 15 min at room temperature in the dark. A 1M H_2_SO_4_ solution was then added to halt colour development. Results are expressed as the optical density proportional to MUC5AC secretion.

**Cytokine Analysis**

Apical washings and basolateral supernatants from unstimulated and IL-13-stimulated PBECs and PNECs (paired) were analyzed using a 27-plex bead array assay (Bio-Plex Pro Human Cytokine 27-plex) (BioRad, UK) as per manufacturers’ instructions. Results are only shown for analytes that fell within the limits of detection. Results are corrected for cell number and expressed as fold change (IL-13 stimulated/unstimulated) on a logarithmic axis.

**Statistical Analysis**

Differences in TEER values were analyzed using repeated measures ANOVA. Total cell number, goblet cell and ciliated cell counts, ELISA data and real time PCR delta delta Ct values were analyzed using paired t-tests and logarithmically transformed where appropriate. Cytokine concentrations were corrected for total cell number and expressed as pg/cell and then the fold change in cytokine secretion following IL-13 stimulation compared with unstimulated cytokine secretion (IL-13-stimulated/unstimulated) was calculated and plotted as fold change on a logarithmic axis. Statistical analysis was performed using JMP® version 9 (SAS, NC, USA).
